# Prognostic implications of superior mesenteric vein/portal vein resection and histologic venous invasion in BR/LA PDAC after neoadjuvant therapy

**DOI:** 10.1007/s00423-026-04145-9

**Published:** 2026-07-24

**Authors:** Jingcheng Zhang, Lei Ren, Carsten Jäger, Alper Doğruöz, Helmut Friess, Ihsan Ekin Demir, Florian Scheufele

**Affiliations:** 1 Department of Surgery, TUM Hospital Rechts der Isar, School of Medicine and Health, Technical University of Munich, Ismaninger Straße 22, 81675 Munich, Bavaria Germany; 2 Department of General Surgery (Gastrointestinal Surgery), The Affiliated Hospital of Southwest Medical University, Luzhou, 646000 China

**Keywords:** Pancreatic cancer, Pancreatectomy, Neoadjuvant therapy, Mesenteric vein, Portal vein

## Abstract

**Background:**

Superior mesenteric vein/portal vein (SMV/PV) resection is often required in borderline resectable or locally advanced pancreatic ductal adenocarcinoma (BR/LA PDAC) after neoadjuvant therapy (NAT), but its prognostic relevance and the role of histopathologic venous invasion remain unclear.

**Methods:**

We retrospectively analyzed BR/LA PDAC patients undergoing pancreatectomy after NAT and compared perioperative and oncologic outcomes between patients requiring SMV/PV resection and those with venous preservation. Subgroup analyses assessed histopathologic venous invasion within the resection cohort. Kaplan–Meier analysis was used to evaluate overall survival (OS) and disease-free survival (DFS), and Cox regression was used to identify factors associated with OS.

**Results:**

Among 117 patients, 65 (55.6%) underwent SMV/PV resection and 52 (44.4%) had venous preservation. Although baseline demographic and clinical characteristics were broadly similar, the resection cohort showed greater local anatomic and operative complexity. Postoperative morbidity and mortality did not differ significantly between groups. Median OS was similar between the SMV/PV resection and preservation groups (16.0 vs. 22.0 months; *P* = 0.965), as was median DFS (10.0 vs. 12.0 months; *P* = 0.843). Within the resection cohort, histopathologically confirmed venous invasion was associated with significantly shorter OS (8.3 vs. 24.0 months; *P* < 0.0001) and DFS (5.0 vs. 17.0 months; *P* = 0.0002), whereas survival was similar between patients with venous preservation and those who underwent resection without venous invasion. R0 resection was associated with longer OS and DFS than R1 resection. In multivariable analysis, R1 resection and histopathologically confirmed SMV/PV invasion were independently associated with poorer OS.

**Conclusions:**

In our cohort, patients requiring SMV/PV resection had outcomes broadly comparable to those with venous preservation despite greater operative complexity. Histopathologic venous invasion and R1 status were associated with worse OS and DFS. These findings support interpreting SMV/PV resection primarily as a marker of local anatomic disease extent rather than an independent adverse prognostic factor.

**Supplementary Information:**

The online version contains supplementary material available at 10.1007/s00423-026-04145-9.

## Introduction

Pancreatic cancer, with approximately 90% of cases being pancreatic ductal adenocarcinoma (PDAC), is the fourth leading cause of cancer-related death in Europe and the seventh worldwide [1]. In Germany, the burden of this aggressive disease continues to grow, with a five-year survival rate of just 11% and nearly identical annual incidence and mortality rates, as approximately 20,200 cases and deaths were recorded in 2020 [2].

Surgical resection remains the only potentially curative treatment for pancreatic cancer. However, only a minority of patients present with upfront resectable disease, whereas a substantial proportion are classified as having borderline resectable (BR) or locally advanced (LA) tumors because of vascular involvement [3]. Over the last decade, neoadjuvant therapy (NAT) has become an established treatment strategy for BR/LA PDAC, aiming to improve systemic disease control, increase the likelihood of resection, and optimize patient selection for surgery [3–7]. In this setting, superior mesenteric vein/portal vein (SMV/PV) resection is frequently required during pancreatectomy.

However, the perioperative and oncologic implications of SMV/PV resection in this setting remain incompletely characterized. Although some studies suggest that venous resection can be performed without a substantial increase in operative risk, others have reported higher morbidity, thrombosis rates, and perioperative mortality [8–12]. In addition, after NAT, treatment-related fibrosis and tissue remodeling may obscure the distinction between persistent tumor infiltration and therapy-induced change, such that the intraoperative need for venous resection does not necessarily correspond to true histopathologic venous invasion [13]. Further characterization of postoperative outcomes and the prognostic relevance of histopathologically confirmed venous invasion in this setting is therefore warranted.

We therefore evaluated perioperative outcomes, overall survival, and prognostic factors in BR/LA PDAC patients undergoing pancreatectomy after NAT, with particular emphasis on SMV/PV resection, histopathologically confirmed venous invasion, and resection margin status.

## Materials and methods

### Study design and patients enrollment

This single-center retrospective cohort study utilized data from a prospectively maintained database. All BR/LA PDAC patients who underwent surgery following NAT at TUM Hospital Rechts der Isar, Technical University of Munich between June 2008 and December 2022 were consecutively included. Patients with incomplete baseline information or those who underwent palliative or tumor-reducing surgery were excluded. Given the retrospective design and the anatomy-driven nature of venous resection, the study was designed as a descriptive comparative and prognostic analysis rather than an assessment of treatment effect.

The study was approved by the Ethics Committee of the Technical University of Munich (approval number 2025-176-S-CB), and all participants provided written informed consent upon inclusion. Regular follow-up was conducted through outpatient visits, telephone calls, and correspondence. This manuscript adheres to the STROBE reporting guidelines.

### Data collection

Preoperative variables, including patient demographics (sex, age), body mass index (BMI), American Society of Anesthesiologists (ASA) classification, unintentional weight loss (UWL) > 10% of body weight, diabetes status, biliary stent, NAT regimen, tumor resectability, carbohydrate antigen 19 − 9 (CA 19 − 9) levels and the angle of tumor-SMV/PV contact, were collected. Intraoperative parameters recorded included the type of surgery performed, SMV/PV resection, operative time, celiac trunk resection, and the number of examined and infiltrated lymph nodes. Pathological outcomes were assessed based on standard pathology reports, including TNM stage, Evans grade, resection margin (R) status, neural invasion, and venous invasion. Postoperative outcomes analyzed included Clavien-Dindo Classification, re-operation, and 30-day mortality, in-hospital, and 90-day mortality.

The assessment of PDAC resectability was primarily based on the latest NCCN guidelines, which classify tumors according to the arterial and venous involvement, including the superior mesenteric artery, celiac axis, hepatic artery, and SMV/PV [3]. The indication for SMV/PV resection was determined by the operating surgeon on the basis of preoperative radiographic findings together with intraoperative evaluation of the relationship between the tumor and the portomesenteric venous axis, particularly when safe dissection from the vein was not considered feasible. Overall survival (OS) was defined as the interval from surgery to death from any cause or last follow-up. Disease-free survival (DFS) was defined as the interval from surgery to first documented recurrence or death from any cause, whichever occurred first. Patients without recurrence or death were censored at the date of last available disease assessment.

The overall postoperative complications were classified according to the Clavien-Dindo Classification System. Histopathologically confirmed SMV/PV invasion was assessed from the final pathology reports in patients who underwent SMV/PV resection. It was defined as microscopic tumor infiltration of the resected portomesenteric venous wall or venous tissue documented by the reporting pathologist. R0 resection is defined as a resection margin of more than 1 mm from tumor cells, including both the surgical resection margins (bile duct, proximal and distal digestive tract, pancreatic stump) and the circumferential resection margins (CRM) including ventral, medial, and retroperitoneal surfaces [14].

### Statistical analysis

All statistical analyses were performed using SPSS 27.0.1.0 and GraphPad Prism 9.0.0. The Shapiro-Wilk test was employed to evaluate the normality of continuous variables. Group comparisons for categorical variables were conducted using the chi-square test or Fisher’s exact test, as appropriate. Bartlett’s test was used to assess the homogeneity of variance for continuous variables. If the assumption of equal variance was met, an independent sample t-test was performed; otherwise, the Mann-Whitney U test was used. Univariate and multivariable Cox proportional hazards models were applied to identify prognostic factors. Kaplan-Meier survival analysis was applied to evaluate OS and DFS, and differences between groups were assessed using the log-rank test. A two-sided P value < 0.05 was considered statistically significant for all analyses.

## Results

A total of 130 patients who underwent surgery following NAT for pancreatic cancer were initially included. Of these, 13 were excluded due to missing baseline data. The final analysis included 117 patients, comprising 57 males and 60 females, with a mean age of 63.03 ± 9.80 years. Among them, 65 patients (55.6%) underwent SMV/PV resection, while 52 patients (44.4%) underwent surgery with SMV/PV preservation.

### Baseline and operative complexity

As shown in Table 1, the two groups were broadly similar with respect to baseline demographic and clinical characteristics, including sex, age, BMI, ASA classification, unintentional weight loss > 10% of body weight, diabetes status, biliary stenting, NAT regimen, and celiac trunk resection. Preoperative CA 19 − 9 levels were numerically higher in the SMV/PV resection group than in the preservation group, although this difference did not reach statistical significance (206.50 vs. 89.00 U/mL, *P* = 0.102). However, several findings suggested greater local anatomic and operative complexity in patients requiring SMV/PV resection. In particular, tumor–SMV/PV contact ≥ 180° was more frequent in the resection group (56.9% vs. 36.5%, *P* = 0.028), and total pancreatectomy was performed significantly more often in this group than in the preservation group (27.0% vs. 7.7%, *P* = 0.004). Operative time also tended to be longer in the resection group across pancreaticoduodenectomy (PD), left pancreatectomy (LP), and total pancreatectomy (TP), although these differences were not statistically significant (PD: *P* = 0.069; LP: *P* = 0.291; TP: *P* = 0.434).

**Table 1:** Pre- and intraoperative characteristics of PDAC patients who underwent surgery following NAT

	Total (*n* = 117)	SMV/PV preservation (*n* = 52)	SMV/PV resection (*n* = 65)		*P* Value ^c^
Sex					0.901
Male	57 (48.7)	25 (48.1)	32 (49.2)		
Female	60 (51.3)	27 (51.9)	33 (50.8)		
Age ^a^	63.03 (9.80)	64.25 (9.34)	62.05 (10.11)		0.289 ^e^
BMI ^a^	24.14 (3.33)	24.35 (3.21)	23.97 (3.55)		0.641 ^e^
ASA					0.581
I-II	64 (54.7)	30 (57.7)	34 (52.3)		
III	53 (45.3)	22 (42.3)	31 (47.7)		
UWL > 10% body weight					0.606
No	99 (84.6)	43 (82.7)	56 (86.2)		
Yes	18 (15.4)	9 (17.3)	9 (13.8)		
Diabetes					0.561
No	95 (81.2)	41 (78.8)	54 (83.1)		
Yes	22 (18.8)	11 (21.2)	11 (16.9)		
Biliary stent					0.473
No	70 (59.8)	33 (63.5)	37 (56.9)		
Yes	47 (40.2)	19 (36.5)	28 (43.1)		
Initial Resectability					0.216
BR-PDAC	38 (32.5)	20 (38.5)	18 (27.7)		
LA-PDAC	79 (67.5)	34 (61.5)	45 (72.3)		
CA 19 − 9 median (U/ml) ^b^	140.00 (31.50–410.00)	89.00 (32.00–231.50)	206.50 (49.50–646.00)		0.102 ^d^
Tumor-SMV/PV contact ≥ 180°	56 (47.9)	19 (36.5)	37 (56.9)		0.028
Initial tumor size (mm) ^b^	40.00 (30.00–45.00)	37.00 (28.75–46.25)	40.00 (30.00–42.00)		0.925 ^d^
NAT regimen					0.236
Folfirinox-based	69 (59.0)	27 (51.9)	42 (64.6)		
Gemcitabine-based	44 (37.6)	22 (42.3)	22 (33.8)		
Others	4 (3.4)	3 (5.8)	1 (1.5)		
NAT cycles ^b^	6.00 (4.00–8.00)	6.00 (4.00–8.00)	6.00 (4.00–8.00)		0.986 ^d^
Operation type					0.004
PD	62 (53.0)	27 (51.9)	35 (53.8)		
LP	33 (28.2)	21 (40.4)	12 (18.5)		
TP	22 (18.8)	4 (7.7)	18 (27.0)		
Operation time (min)					
PD ^b^	471.50 (400.50–557.25)	444.00 (386.00–506.00)	508.00 (405.00–593.00)		0.069 ^d^
LP ^b^	300.00 (245.50–401.50)	300.00 (245.50–360.00)	340.50 (252.50–587.75)		0.291 ^d^
TP ^b^	472.00 (409.75–562.50)	437.50 (413.25–473.75)	491.50 (399.25–573.00)		0.434 ^d^

Values in parentheses are percentages unless indicated otherwise; ^a^ values are mean (s.d.); ^b^ values are median (i.q.r.). *PDAC* pancreatic ductal adenocarcinoma, *NAT* neoadjuvant therapy, *SMV* superior mesenteric vein, *PV* portal vein, *BMI* body mass index, *ASA* American Society of Anesthesiologists, *UWL* unintentional weight loss, *BR-PDAC* borderline resectable PDAC, *LA-PDAC* locally advanced PDAC, *CA 19 − 9* Carbohydrate-Antigen 19 − 9, *PD* pancreaticoduodenectomy, *LP* left pancreatectomy, *TP* total pancreatectomy. ^c^ Chi-square test or Fisher’s exact test, except ^d^ Mann–Whitney U test and ^e^ t test

Given the extended inclusion period, we additionally assessed potential treatment-era effects. Patients were stratified into an earlier era (2008–2015; *n* = 40) and a later era (2016–2022; *n* = 77). As shown in Supplementary Table S1, NAT regimens changed over time, with a shift from more heterogeneous gemcitabine-based approaches in earlier years toward more frequent FOLFIRINOX-based treatment in the later era. Although patient and treatment characteristics differed between eras, treatment era was not significantly associated with OS or DFS.

### Surgical and histopathological outcomes

Comparisons of surgical and pathological outcomes between the two groups are presented in Table 2. Patients in the SMV/PV resection group had a significantly higher number of examined lymph nodes [31.00 (24.00–40.00) vs. 19.50 (13.25–29.00); *P* < 0.001], more infiltrated lymph nodes [2.00 (0.00–3.00) vs. 0.00 (0.00–1.00); *P* = 0.010], and a higher ratio of infiltrated to examined lymph nodes [0.04 (0.00–0.13) vs. 0.00 (0.00–0.06); *P* = 0.032]. While the distribution of pathological N stage did not differ significantly between groups, there was a trend toward a higher proportion of N1/N2 disease in the SMV/PV resection group (67.7% vs. 50.0%, *P* = 0.060). Among the 65 patients who underwent SMV/PV resection, histopathologically confirmed SMV/PV invasion was identified in 22 patients (33.8%). No significant differences were observed in pathological tumor size, T stage, Evans grade, R status, or the presence of neural, venous, or lymphatic vessel invasion (*P* > 0.05 for all). Likewise, postoperative outcomes, including delayed gastric emptying (DGE), postpancreatectomy hemorrhage (PPH), wound infection, major complications (Clavien-Dindo classification IV-V), reoperation, and in-hospital, 30-day, and 90-day mortality, did not differ significantly between groups (*P* > 0.05 for all).

**Table 2:** Pathological and surgical outcomes of PDAC patients undergoing surgery following neoadjuvant therapy

	Total (*n* = 117)		SMV/PV preservation (*n* = 52)	SMV/PV resection (*n* = 65)	*P* Value^b^
Examined lymph nodes ^a^	28.00 (18.00–37.00)		19.50 (13.25–29.00)	31.00 (24.00–40.00)	< 0.001 ^c^
Infiltrated lymph nodes ^a^	1.00 (0.00–3.00)		0.00 (0.00–1.00)	2.00 (0.00–3.00)	0.010 ^c^
Ratio of infiltrated/examined lymph nodes ^a^	0.03 (0.00–0.09)		0.00 (0.00–0.06)	0.04 (0.00–0.13)	0.032 ^c^
Pathological tumor size (mm) ^a^	30.00 (22.00–40.00)		28.50 (22.25–36.50)	30.00 (21.00–42.00)	0.579 ^c^
T stage: yT3-4	42 (35.9)		17 (32.7)	25 (38.5)	0.565
N stage: yN1-2	70 (59.8)		26 (50.0)	44 (67.7)	0.060
Evans grade: ≥2b	34 (29.1)		16 (30.8)	18 (27.7)	0.927
R status: R1	71 (60.7)		33 (63.5)	38 (58.5)	0.704
Neural invasion (+)	84 (71.8)		37 (71.2)	47 (72.3)	1.000
Microscopic venous invasion (+)	34 (29.1)		12 (23.1)	22 (33.8)	0.202
Lymphatic vessels invasion (+)		48 (41.0)	17 (32.7)	31 (47.7)	0.101
CDC: IV-V		14 (12.0)	6 (11.5)	8 (12.3)	0.660
DGE	16 (13.7)		6 (11.5)	10 (15.4)	0.547
PPH	17 (14.5)		5 (9.6)	12 (18.5)	0.177
Wound infection	11 (9.4)		2 (3.8)	9 (13.8)	0.128
Re-operation	12 (10.3)		5 (9.6)	7 (10.8)	0.838
In-hospital mortality	3 (2.6)		1 (1.9)	2 (3.1)	0.695
30-day mortality	2 (1.7)		1 (1.9)	1 (1.5)	0.873
90-day mortality	9 (7.7)		4 (7.7)	5 (7.7)	1.000

Values are presented as n (%) unless indicated otherwise; ^a^ values are median (i.q.r.). *PDAC* pancreatic ductal adenocarcinoma, *NAT* neoadjuvant therapy, *SMV* superior mesenteric vein, *PV* portal vein, *CDC* Clavien Dindo classification, *DGE* delayed gastric emptying, *PPH* postpancreatectomy hemorrhage. ^b^ Chi-square test or Pearson Fisher’s exact test, except ^c^ Mann–Whitney U test

### Oncologic outcomes

**Fig. 1:**
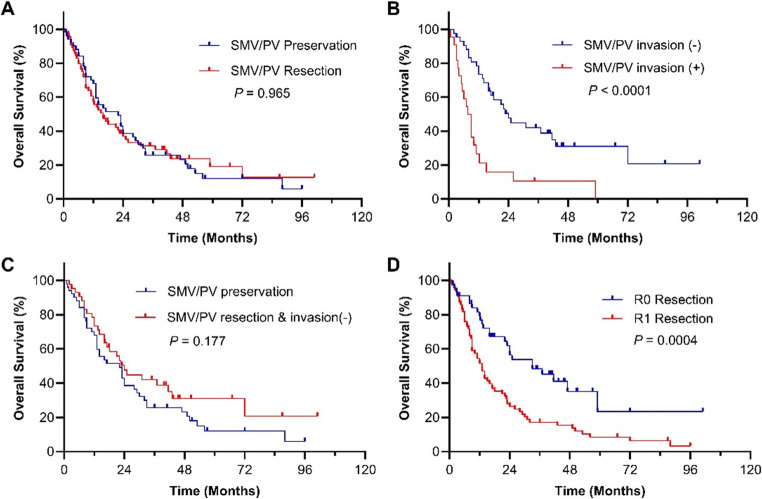
Kaplan-Meier overall survival curves for patients undergoing surgery following neoadjuvant therapy. (**a**) SMV/PV resection vs. preservation. (**b**) SMV/PV invasion negative vs. positive. (**c**) SMV/PV preservation vs. resection with invasion negative. (**d**) R0 vs. R1 resection

The median follow-up duration for the entire cohort was 15.5 months (range, 1–101 months). Among surviving patients, the median follow-up was 39.8 months, compared with 13.0 months among those who had died. The median postoperative overall survival (OS) for PDAC patients who underwent surgery following NAT was 17.0 months (95% confidence interval [CI]: 10.47–23.53), with estimated 2- and 5-year survival rates of 37.6% and 14.5%, respectively. In the crude survival analysis, OS did not differ significantly between the SMV/PV preservation group and the SMV/PV resection group (median: 22.00 months, 95% CI: 13.00–28.00 vs. 16.00 months, 95% CI: 11.00–24.00; *P* = 0.965; Fig. 1A). However, within the SMV/PV resection cohort, histopathologically confirmed venous invasion was associated with significantly shorter OS: patients without venous invasion had a median OS of 24.00 months (95% CI: 16.00–43.00), compared with 8.30 months (95% CI: 4.00–11.00) in those with confirmed invasion (*P* < 0.0001; Fig. 1B). In addition, patients who underwent SMV/PV resection without histopathologic venous invasion had OS similar to that of patients with venous preservation (22.00 vs. 24.00 months; *P* = 0.177; Fig. 1C). Margin status was also strongly associated with survival: patients who achieved R0 resection had significantly longer OS than those with R1 resection (median: 33.00 months, 95% CI: 22.00–59.00 vs. 13.00 months, 95% CI: 9.00–17.00; *P* = 0.0004; Fig. 1D).

**Fig. 2:**
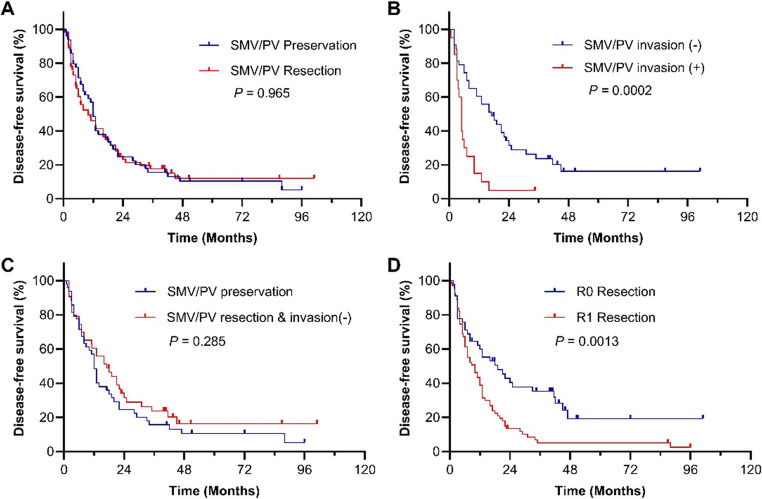
Kaplan-Meier disease-free survival curves for patients undergoing surgery following neoadjuvant therapy. (**a**) SMV/PV resection vs. preservation. (**b**) SMV/PV invasion negative vs. positive. (**c**) SMV/PV preservation vs. resection with invasion negative. (**d**) R0 vs. R1 resection

Disease-free survival (DFS) showed a similar pattern. The median DFS for the entire cohort was 11.05 months, with estimated 2- and 5-year DFS rates of 23.9% and 7.5%, respectively. DFS did not differ between the SMV/PV preservation and SMV/PV resection groups (median DFS, 12.00 vs. 10.00 months; *P* = 0.843; Fig. 2A). However, patients with histopathologically confirmed SMV/PV invasion had significantly shorter DFS than those without confirmed venous invasion (median DFS, 5.00 vs. 17.00 months; *P* = 0.0002; Fig. 2B). In patients without histopathologic venous invasion, DFS was not significantly different between those who underwent SMV/PV resection and those who underwent SMV/PV preservation (median DFS, 17.00 vs. 12.00 months; *P* = 0.285; Fig. 2C). R1 resection was associated with inferior DFS compared with R0 resection (median DFS, 10.00 vs. 19.00 months; *P* = 0.0013; Fig. 2D).

### Prognostic factors for the PDAC patients undergoing surgery following NAT

As shown in Table 3, univariable Cox regression analysis identified ASA classification III/IV, neural invasion, histopathologically confirmed SMV/PV invasion, and R1 resection status as factors associated with poorer overall survival in PDAC patients who underwent surgery following NAT. A gemcitabine-based NAT regimen also showed a trend toward worse survival (*P* = 0.082) and was therefore entered into the multivariable model. In the final multivariable analysis, R1 resection (HR 1.989, 95% CI: 1.217–3.251, *P* = 0.006) and histopathologically confirmed SMV/PV invasion (HR 2.508, 95% CI: 1.480–4.250, *P* < 0.001) remained independently associated with poorer overall survival.

**Table 3:** Univariate and multivariable analyses of prognostic factors in PDAC patients underwent surgery following neoadjuvant therapy

	Univariate HR (95% CI)	*p* Value	Multivariable HR (95% CI)	*p* Value
ASA classification III/IV	1.583 (1.042–2.406)	0.031	1.182 (0.754–1.854)	0.466
NAT regimen				
Folfirinox-based	1			
Gemcitabine-based	1.470 (0.952–2.269)	0.082	1.292 (0.831–2.009)	0.255
Others	1.217 (0.423–3.500)	0.715		
R1 status	2.258 (1.416–3.599)	< 0.001	1.989 (1.217–3.251)	0.006
Neural invasion (+)	1.958 (1.175–3.261)	0.010	1.445 (0.864–2.468)	0.177
SMV/PV invasion (+)	2.691 (1.615–4.485)	< 0.001	2.508 (1.480–4.250)	< 0.001

*PDAC* pancreatic ductal adenocarcinoma, *HR* hazard ratio, *CI* confidence interval, *SMV* superior mesenteric vein, *PV* portal vein

## Discussion

There is now broad consensus that selected patients with pancreatic cancer involving major vascular structures may still achieve meaningful long-term survival through multimodal therapy followed by resection at experienced high-volume centers [15]. In this setting, SMV/PV resection is frequently required because of tumor–vessel anatomy and the inability to establish a safe dissection plane. However, in BR/LA PDAC patients undergoing pancreatectomy after NAT, perioperative and oncologic outcomes after SMV/PV resection remain incompletely characterized, and the extent to which these outcomes are influenced by underlying histopathologic venous invasion remains unclear.

In the present study, we analyzed 117 BR/LA PDAC patients who underwent surgery following NAT, of whom 55.6% required SMV/PV resection. Although many baseline demographic and clinical variables were broadly similar between the groups, the resection cohort demonstrated features consistent with greater local anatomic and operative complexity, including a higher rate of tumor–SMV/PV contact ≥ 180°, a higher proportion of TP, and a greater nodal tumor burden on pathological assessment. These findings suggest that patients undergoing SMV/PV resection represented a biologically and anatomically more advanced subgroup, which is in line with previous studies [8, 10].

With regard to perioperative outcomes, the rates of major complications, 30-day mortality, in-hospital mortality, and 90-day mortality in the SMV/PV resection group were 12.7%, 1.6%, 3.2%, and 7.9%, respectively. These results are broadly in line with a large multicenter cohort study of 2,265 PDAC patients, including 694 who underwent SMV/PV resection, in which 30-day and 90-day mortality rates were 1.5% and 6.3%, respectively [9]. In our cohort, postoperative complications did not differ significantly between patients with and without SMV/PV resection. However, this finding should be interpreted cautiously, as the two groups differed in operative complexity and the study lacked granular data on venous resection type and reconstruction method, both of which are known to influence morbidity. Prior studies have shown higher benchmark values for major complications and in-hospital mortality in pancreatoduodenectomy with SMV/PV resection than in standard pancreatoduodenectomy without vascular resection [16, 17]. Similarly, patients undergoing left pancreatectomy with venous resection have been reported to experience higher major morbidity than those without venous resection (40.5% vs. 22.3%, *p* < 0.001) [12], although whether this reflects the resection itself or the underlying extent of disease remains debated. A single-center propensity score-matched study found comparable morbidity between standard DP and DP with SMV/PV resection [18]. Additionally, postoperative morbidity and mortality rates vary significantly based on the type of venous reconstruction performed. In PD patients, segmental vein resection was associated with a higher incidence of major complications compared to wedge resection (39% vs. 20%, *P* < 0.001); in the same report, among patients with venous resection following NAT, major morbidity rates were reported to be as high as 52%, underscoring the potential additive risk of combining complex vascular procedures with neoadjuvant treatment [11].

Indeed, although numerous large-scale multicenter studies and meta-analyses have demonstrated the overall safety of pancreatectomy following NAT, conflicting evidence persists [19, 20]. For instance, a large-scale analysis of 6,936 patients found that preoperative chemoradiotherapy was associated with a significantly higher 90-day mortality rate compared to chemotherapy alone (6.4% vs. 3.6%, *P* < 0.001) [21]. One proposed mechanism is that neoadjuvant radiotherapy may induce vascular wall changes, potentially increasing the risk of intraoperative bleeding; however, this hypothesis has not been consistently validated [22]. Despite these concerns, our study provides preliminary evidence supporting the feasibility and acceptable safety profile of SMV/PV resection in BR/LA PDAC patients following NAT. However, further large-scale, multicenter studies with detailed subgroup analyses are necessary to better characterize the surgical outcome associated with venous resection in this special clinical context.

In our cohort, survival outcomes were comparable between the SMV/PV resection and preservation groups, aligning with previous meta-analyses that suggest synchronous SMV/PV resection does not inherently compromise long-term prognosis in patients undergoing pancreatectomy for PDAC [23]. At the same time, more recent studies have reported inferior survival outcomes in patients undergoing SMV/PV resection, often attributing this to more advanced tumor stage and lower R0 resection rates rather than the resection itself [8, 9]. In the present cohort, the lack of a significant crude survival difference should therefore not be interpreted as evidence of a treatment effect of venous resection, but rather as an observation that patients requiring venous resection achieved survival broadly comparable to those with venous preservation despite more complex local disease.

A particularly important finding of this study is that, within the SMV/PV resection cohort, histopathologically confirmed venous invasion was associated with markedly shorter survival, whereas patients who underwent venous resection without confirmed invasion had survival similar to that of patients with venous preservation. This suggests that true venous wall invasion may better capture the adverse biological behavior underlying worse prognosis. Our findings are consistent with earlier reports questioning whether negative venous invasion should be interpreted as a missed opportunity for routine resection. In 2013, Turrini et al. reported a survival benefit in patients without venous invasion who underwent SMV/PV resection [24], raising the possibility that routine resection might improve oncologic outcomes. However, later and larger studies failed to confirm a survival advantage for routine venous resection in the absence of histologically proven invasion [25, 26]. In the NAT setting, Chae et al. likewise found no survival difference between SMV/PV resection and preservation after pancreaticoduodenectomy, although the resection group had a higher incidence of early postoperative thrombosis [27]. Taken together with our data, these findings support a more restrained interpretation: venous resection should be regarded primarily as an anatomy-driven operative decision, while histopathologic venous invasion provides more meaningful prognostic stratification.

Our data also reinforce the central importance of radical resection. Patients who achieved R0 resection had significantly longer survival than those with R1 resection, with longer OS (median, 33 versus 13 months) and improved DFS (median, 19 versus 10 months). Given that median survival in patients with BR/LA PDAC receiving only palliative therapy is typically less than one year [28], this finding highlights the potential value of multimodal treatment followed by surgery when oncologic clearance can be achieved. In this context, margin status remains a more actionable prognostic marker than venous resection status alone.

As a single-center retrospective study, selection bias is inherently unavoidable. Moreover, the extended study period may introduce temporal heterogeneity, given the evolving NAT protocols, surgical decision-making, perioperative management, and pathological assessment over time. Although treatment era was not significantly associated with OS or DFS in our exploratory analysis, residual temporal heterogeneity cannot be fully excluded. The heterogeneity between the SMV/PV resection and preservation groups further limits the direct comparability of outcomes. Although multivariable Cox regression was used to adjust for selected measured prognostic factors, no propensity score matching was performed; therefore, residual confounding and baseline imbalance cannot be excluded. Additionally, the relatively small sample size precluded more granular subgroup analyses, thereby reducing the statistical power and limiting the generalizability of the results.

## Conclusions

In BR/LA PDAC patients undergoing pancreatectomy after NAT, those requiring SMV/PV resection achieved perioperative and survival outcomes broadly comparable to those with venous preservation, despite features of greater local anatomic and operative complexity. Histopathologically confirmed SMV/PV invasion and R1 resection, rather than venous resection status, were associated with worse survival. These findings support interpreting SMV/PV resection primarily as a marker of local disease extent and operative complexity, while emphasizing the prognostic importance of true venous invasion and margin clearance in this setting.

## Supplementary Information

Below is the link to the electronic supplementary material.

Supplementary Material 1 (DOCX 18.1 KB )

## Data Availability

The datasets analyzed during the current study are available from the corresponding author on reasonable request.
