# Supplementary material for: Prognostic implications of superior mesenteric vein/portal vein resection and histologic venous invasion in BR/LA PDAC after neoadjuvant therapy
Source: Langenbecks Arch Surg. 2026 Jul 24;411(1):200. doi: 10.1007/s00423-026-04145-9 (PMC13400680; doi:10.1007/s00423-026-04145-9)
Supplement: Supplementary file 1 — Supplementary Material 1 (DOCX 18.1 KB ) [file 423_2026_4145_MOESM1_ESM.docx]

**Supplementary Table 1**. Distribution of NAT regimens and outcomes according to treatment era.

|  | **2008–2015 (n=40)** | **2016–2022 (n=77)** | ***P* value** |
| --- | --- | --- | --- |
| Initial Resectability |  |  | 0.041 |
| BR-PDAC | 18 (45.0) | 20 (26.0) |  |
| LA-PDAC | 22 (55.0) | 57 (74.0) |  |
| NAT regimen |  |  | 0.009 |
| Folfirinox-based | 19 (47.5) | 50 (64.9) |  |
| Gemcitabine-based | 17 (42.5) | 27 (35.1) |  |
| Others | 4 (10.0) | 0 (0) |  |
| NAT regimen, detailed category |  |  | Descriptive |
| FOLFIRINOX-based only | 18 (45.0) | 38 (49.4) |  |
| Sequential or mixed systemic regimens including FOLFIRINOX | 1 (2.5) | 9 (11.7) |  |
| FOLFIRINOX-based, not further specified | 0 (0) | 3 (3.9) |  |
| Gemcitabine/nab-paclitaxel | 3 (7.5) | 12 (15.6) |  |
| GemOX or other gemcitabine/platinum-based regimens | 7 (17.5) | 0 (0) |  |
| Gemcitabine monotherapy or gemcitabine/erlotinib | 7 (17.5) | 1 (1.3) |  |
| Gemcitabine-based, not further specified | 0 (0) | 14 (18.2) |  |
| Fluoropyrimidine-based chemoradiotherapy | 1 (2.5) | 0 (0) |  |
| Other non-gemcitabine/non-FOLFIRINOX systemic regimens | 2 (5.0) | 0 (0) |  |
| Complex or unclear chemoradiotherapy regimen | 1 (2.5) | 0 (0) |  |
| Radiotherapy component, yes | 9 (22.5) | 15 (19.5) |  |
| NAT cycles ^a^ | 4.00 (3.00-6.00) | 4.00 (3.60-6.00) | 0.716 ^b^ |
| SMV/PV resection | 13 (32.5) | 52 (67.5) | <0.001 |
| R1 resection | 32 (80) | 39 (50.6) | 0.003 |
| Median OS, months (95% CI) | 18.00 (8.70-27.30) | 16.00 (6.09-25.91) | 0.832 ^c^ |
| Median DFS, months (95% CI) | 13.00 (11.80-14.20) | 8.00 (4.20-11.80) | 0.557 ^c^ |

Values in parentheses are percentages unless indicated otherwise; ^a^ values are median (i.q.r.). NAT, neoadjuvant therapy; PDAC, pancreatic ductal adenocarcinoma; BR-PDAC, borderline resectable PDAC; LA-PDAC, locally advanced PDAC; OS, overall survival; DFS, disease-free survival. Chi-square test or Pearson Fisher's exact test, except ^b^ Mann–Whitney U test and ^c^ Log rank test.
